# Poly(HydroxyButyrate-co-HydroxyValerate) (PHBHV) Nanocarriers for Silymarin Release as Adjuvant Therapy in Colo-rectal Cancer

**DOI:** 10.3389/fphar.2017.00508

**Published:** 2017-08-02

**Authors:** Ionut-Cristian Radu, Ariana Hudita, Catalin Zaharia, Paul O. Stanescu, Eugenia Vasile, Horia Iovu, Miriana Stan, Octav Ginghina, Bianca Galateanu, Marieta Costache, Peter Langguth, Aristidis Tsatsakis, Kelly Velonia, Carolina Negrei

**Affiliations:** ^1^Advanced Polymer Materials Group, University Politehnica of Bucharest Bucharest, Romania; ^2^Department of Biochemistry and Molecular Biology, University of Bucharest Bucharest, Romania; ^3^Department of Bioresources and Polymer Science, University Politehnica of Bucharest Bucharest, Romania; ^4^Department of Toxicology, Faculty of Pharmacy, Carol Davila University of Medicine and Pharmacy Bucharest, Romania; ^5^Department of Surgery, Sf. Ioan Emergency Clinical Hospital Bucharest, Romania; ^6^Department II, Faculty of Dental Medicine, Carol Davila University of Medicine and Pharmacy Bucharest Bucharest, Romania; ^7^Research Institute of University of Bucharest, University of Bucharest Bucharest, Romania; ^8^Department of Pharmaceutical Technology and Biopharmaceutics, Institute of Pharmacy, Johannes Gutenberg-University Mainz, Germany; ^9^Department of Toxicology and Forensic Sciences, Faculty of Medicine, University of Crete Heraklion, Greece; ^10^Department of Materials Science and Technology, University of Crete Heraklion, Greece

**Keywords:** nanocarriers, drug delivery, colo-rectal cancer, Silymarin, Poly(HydroxyButyrate-co-HydroxyValerate) (PHBHV)

## Abstract

The aim of this study was to address one of the major challenges of the actual era of nanomedicine namely, the bioavailability of poorly water soluble drugs such as Silymarin. We developed new, biodegradable, and biocompatible nanosized shuttles for Silymarin targeted delivery in colon-cancer cells. The design of these 100 nm sized carrier nanoparticles was based on natural polymers and their biological properties such as cellular uptake potential, cytotoxicity and 3D penetrability were tested using a colon cancer cell line (HT-29) as the *in vitro* culture model. Comparative scanning electron microscopy (SEM) and atomic force microscopy (AFM) measurements demonstrated that the Silymarin loaded Poly(3-HydroxyButyrate-co-3-HydroxyValerate) (PHBHV) nanocarriers significantly decreased HT-29 cells viability after 6 and 24 h of treatment. Moreover, *in vivo-*like toxicity studies on multicellular tumor spheroids showed that the Silymarin loaded PHBHV nanocarriers are able to penetrate 3D micro tumors and significantly reduce their size.

## Introduction

The past few years have witnessed major developments in nanoscience and nanotechnology with great potential in powering new diagnostic and therapeutic tools for nanomedicine. Under this umbrella, during the 2000s different nanosized engineered therapeutics and imaging agents have started to evolve (Duncan and Gaspar, 2011), with nanoparticles attracting much attention for biomedical applications primarily due to their new, intriguing properties that are mainly attributed to their large surface to mass ratio. In fact, simply by the virtue of their size, nanoparticles display unique features that distinguish them from bulk materials such as the ability to adsorb, shield and carry compounds, unique chemical reactivity, energy absorption, and biological mobility (Murthy, 2007). However, many environmental and societal challenges, particularly regarding their toxicity must be overcome (Piperigkou et al., 2016).

Many nanoscale systems developed to serve imaging applications improve traditional imaging methods such as modern magnetic resonance imaging (Duncan and Gaspar, 2011; Duncan and Vicent, 2013), but the major interest nowadays resides in the development of targeted drug delivery shuttles (Yin et al., 2014). More specifically, nanoparticles are being studied as promising active vectors due to their capacity to encapsulate and deliver drugs. These carriers offer several new advantages over classic administration of drugs, such as protection of the encapsulates (drugs, vitamins, antioxidants, proteins, and lipids) against degradation, improvement of the therapeutic concentration of both water soluble and insoluble bioactive compounds, controlled retention time, bioavailability and, most importantly, decrease of toxicity (Pinto Reis et al., 2006; Mora-Huertasa et al., 2010; Noronhaa et al., 2013). Furthermore, it has been shown that nanoparticles are perfect match for tumor targeting due to their ability to penetrate the leaky neo-vasculatures and accumulate as a result of the poor lymphatic drainage of solid tumors (Fang et al., 2011; Maeda et al., 2013). In this view, an important number of nanoparticle formulations of different compositions are being evaluated as anticancer drug delivery systems and are currently in clinical trials for a large spectrum of medical applications, including the targeting of solid tumors (Hrkach et al., 2012; Veiseh et al., 2015; Kim et al., 2016; Kuskov et al., 2016).

With respect to materials for nanoparticle design, literature reports various sources like: natural compounds (phospholipids, lipids, lactic acid, dextran, gelatin, chitosan, silk), synthetic polymers, silica, metals, etc. (Cannizzaro and Langer, 1999). Polymeric nanoparticles are mostly solid colloidal nanospheres or nanocapsules, often stabilized by surfactants. Biodegradable polymers in particular have been widely used for the development of nanosized drug delivery systems owing their huge potential as drug carriers mainly to their ability to degrade and their capacity for controlled drug release (Mora-Huertas et al., 2012). Drug loaded polymeric nanocarriers can be obtained either directly from the polymerization of a large variety of monomers in the presence of the drug, or from preformed polymers via solvent evaporation (Harmia et al., 1986; Saxena et al., 2005), emulsification (Cascone et al., 2002), reverse phase preparation (Gupta et al., 2004), coacervation (Leo et al., 1997), or nanoprecipitation (Galindo-Rodriguez et al., 2004; Alshamsan, 2014). Polymer nanocapsules more specifically, are vesicular systems with a typical core-shell structure, where the core can be a polymeric reservoir or an inner liquid while the shell consists of a polymeric membrane or coating. Drugs encapsulated within such nanocapsules are usually loaded in the core either by dissolving or by dispersing the drug in the polymeric reservoir, while in several cases a gradient of the drug might also be adsorbed on the shell. In contrast, the nanospheres have a polymeric matrix-like structure with the encapsulates either dispersed into the polymeric matrix or adsorbed to the nanospheres surface (Pinto Reis et al., 2006; Mora-Huertasa et al., 2010).

Beyond advantages stemming from their nanoscale size, polymeric nanoparticles are also characterized by size distribution, surface charge, surface adhesion, interior porosity, and drug encapsulation efficiency and stability (Gratton et al., 2008; Kumari et al., 2010). Both surface charge and chemistry are crucial for biomedical applications and more specifically for tailoring the interaction of nanoparticles with blood components (proteins, antibodies, small molecules, etc.) as well as for controlling factors such as adherence and interaction with cellular membranes. Furthermore, the surface chemistry particularities may enable the nanoparticle's stealth properties against the natural defense system of the body (mononuclear phagocytic system).

Poly(3-HydroxyButyrate-co-3-HydroxyValerate) (PHBHV) is a natural polyester produced from renewable sources by a great variety of microorganisms. PHBHV is in fact a copolymer of the highly popular poly(hydroxybutyrate) which is extensively tested as implantable biomaterial in medical studies and exhibits excellent biocompatibility and biodegradability properties (Ojumu et al., 2004; Freier, 2006; Shishatskaya et al., 2016). Due to its high solubility in chloroform or dichloromethane and poor solubility in other solvents (Poletto et al., 2007, 2008), PHBHV can easily form nanoparticles through the emulsification-solvent evaporation method (Pich et al., 2006; Poletto et al., 2008). In previous studies, PHBHV nanoparticles were obtained via emulsification-diffusion in a chloroform/ethanol solvent mixture and by an oil in water emulsion (Pich et al., 2006; Poletto et al., 2008).

The anti-neoplastic potential of natural agents has been widely investigated in relation to their action on various steps of the carcinogenesis process (Pratheeshkumar et al., 2012). Research in this area is largely focused on the defining features of cancer, i.e., apoptosis, cell cycle, angiogenesis, invasion, and eventually metastasis through studies aiming to indicate the anti-cancer efficacy of plant chemo-preventive agents by investigating their capacity to act on one or several of the processes involved (Agarwal et al., 2006). In this view, recent studies revealed Silymarin as a powerful and promising complex against Colo-rectal cancer occurrence and progress, though its mechanism of action remains to be clarified. Silymarin is extracted from the seeds of the plant *Silybummarianum* (L.) and the so-called Silymarin complex is a mixture of flavonolignans (65–80%), fatty acids (ca. 20–35%), flavonoids, and various other polyphenolic compounds in smaller amounts. The Silymarin complex includes silybin (silibinin) as major component, silychristin, isosilybin, and silydianin among the other flavonolignans, as well as the flavonoid taxifolin (Szilági et al., 1981; Lee and Liu, 2003; Kroll et al., 2007; MacKinnon et al., 2007). Silymarin has been used for more than 2000 years for the improvement of liver conditions and against hepato-toxicity in general (Post-White et al., 2007). Studies on its action on the liver have determined anti-inflammatory, anti-lipid peroxidative, anti-oxidative, anti-fibrotic pharmacological effects as well as mechanisms for membrane stabilization, immuno-modulation, and liver regeneration (Cetinkunar et al., 2015). Research on Silibinin's possible mechanism of action as a chemo-preventive agent has brought about evidence of its ability to interfere with expression of apoptosis-related proteins and cell-cycle regulating factors, thus acting as a modulator of the cell survival–apoptosis imbalance. The anti-metastatic and anti-inflammatory effect of Silymarin has been explained by its potential to modulate specific proteins while, pre-clinical and clinical research to date have confirmed the mechanism of Silymarin metabolism, and elucidated pharmacokinetics and pharmacodynamics relevant to its anti-neoplastic use (Kim et al., 2003; Hoh et al., 2006; Wu et al., 2006).

*In vivo* studies have indicated a dependence of Silymarin flavonolignans effectiveness on both the presence of on-site therapeutic concentrations as well as on bioavailability. Nevertheless, due to Silymarin's poor water solubility, preclinical and clinical pharmacokinetic studies have accordingly shown minimal (in the range of ng/ml) plasma concentrations after oral administration of Silymarin extract in powder form. The same studies have further indicated the possibility to improve Silybin bioavailability by administration combined with phosphaditylcholine (Barzaghi et al., 1990).

Considering the aforementioned studies, we aimed to increase Silymarin's bioavailability via its encapsulation within novel biocompatible and biodegradable nanocarriers. We present here the construction as well as the characterization and *in vitro* cytotoxicity evaluation of a newly developed PHBHV nanocarrier loaded with Silymarin and designed for prospective use in Colo-rectal cancer therapy.

## Methods

### Formation of PHBHV nanoparticles and Silymarin loading

**Figure 1:**
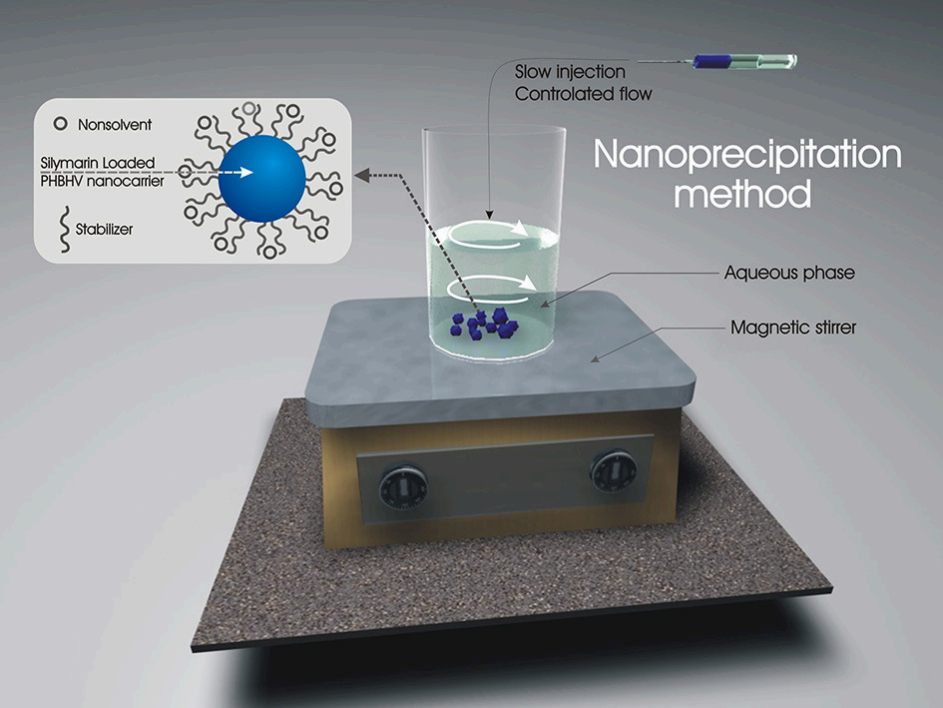
Set-up and experimental procedure for the preparation of nanoparticles by the nanoprecipitation method.

PHBHV with molecular weight of 67,000 g/mol and 2% hydroxyvalerate content was obtained from Good Fellow, polyvinyl alcohol (PVA) with molecular weight of 88,000 g/mol, 88% hydrolyzed, and chloroform were supplied by Sigma Aldrich. Based on literature reports (Pich et al., 2006; Poletto et al., 2007, 2008; Kumari et al., 2010), in this paper, we optimized the formation of nanoparticles by nanoprecipitation of a PHBHV solution in a PVA aqueous solution (Figure 1).

Briefly, a PHBHV solution in chloroform and a PVA aqueous solution of fixed concentrations were initially prepared. The polymer solution was added dropwise to the PVA stabilizer solution over a period of 5 h under controlled solvent flow conditions (1 mL/h) and vigorous stirring. The resulting particle size was studied by variation of crucial factors such as polymer concentration (organic phase), stabilizer concentration (aqueous phase), and ratio between the two phases. In order to obtain smaller nanoparticle size, polymer concentration was initially varied in the range 1–5% w/w while the other parameters were kept constant. The effect of stabilizer concentration was also studied by varying its concentration in the same manner (1–5% w/w) and finally the effect of the ratio between the organic and the aqueous phase was studied in the range of 1:5–1:15 (organic: aqueous phase volume). The obtained nanoparticles were subsequently centrifuged and extensively washed for PVA removal. Silymarin exhibits excellent solubility in chloroform and was consequently loaded in the nanocarriers by its direct solubilization in the polyester solution.

The amount of the drug encapsulated within the PHBHV nanoparticles was investigated by UV-VIS spectrophotometry. Silymarin presents two absorbance peaks in the UV-VIS region at 285 and 325 nm. In order to determine Silymarin content, the UV-VIS absorbance was measured at 325 nm using a Shimadzu UV-VIS-NIR spectrophotometer. The drug content (DC) and the encapsulation efficiency (EE) were determined using the following equations:

$$\begin{array}{l} \text{DC}\left(\text{\%}\right)=\frac{weight\;of\;Silymarin\;entrapped\;in\;the\;nanoparticles}{total\;weight\;of\;the\;Silymarin\;loaded\;nanoparticles}\\ \times 100\\ \text{EE}\left(\%\right)=\frac{weight\;of\;Silymarin\;entrapped\;in\;the\;nanoparticles}{total\;weight\;of\;the\;Silymarin\;used}\\ \times 100 \end{array}$$

### Silymarin drug release

The *in vitro* release of Silymarin from the polyester nanoparticles was evaluated for a period of 48 h. More specifically, Silymarin loaded nanoparticles (10 mg) were added in 10 mL of phosphate buffered saline, PBS (0.01 M, pH 7.4), and incubated in a precision water bath (orbital mixer Benchmark Scientific) at 300 rpm and 37.0 ± 0.5°C. At defined time points, 5 mL of supernatant was collected and an equivalent amount of fresh PBS at 37.0 ± 0.5°C was added to maintain the total volume of the sample stable. Drug release was studied for 48 h and samples were collected every 15 min for the first hour, every 30 min until 4 h, and every 60 min until the end of the experimental time. The Silymarin release from the PHBHV nanocarriers was evaluated by UV-VIS spectroscopy.

### Characterization of the PHBHV nanocarriers by scanning electronic microscopy (SEM) and atomic force microscopy (AFM)

#### Scanning electron microscopy (SEM)

Nanoparticles size, size distribution as well as morphology were investigated using SEM. Dried PHBHV nanoparticles were analyzed after gold-coating using a Quanta Inspect F SEM device equipped with a field emission gun (FEG) with 1.2 nm resolution and with an X-ray energy dispersive spectrometer (EDS).

#### Atomic force microscopy (AFM)

AFM was employed to determine not only the PHBHV interaction with cells but also to investigate the potential uptake of the nanoparticles by the cells. The mean size and size distribution of the PHBHV nanocarriers were measured in contact mode by a multimodal AFM (Agilent 5500) equipped with a controller AC Mode III with a scanning capability of 90 × 90 μm in the xy direction and of 7 μm in the z direction.

### *In vitro* cytotoxicity assessment of the PHBHV nanocarriers

#### Cell culture model

The HT-29 human colon adenocarcinoma cell line (American Type Culture Collection) was used as cellular model in this study. Cells were cultured at 37°C as 2D monolayers under a humidified atmosphere of 5% CO_2_, in Dulbecco's modified Eagle's Medium (DMEM) supplemented with 10% fetal bovine serum (FBS) and 1% penicillin–streptomycin. After reaching 80% confluence, the cells were successively subcultured by trypsin treatment and the medium was refreshed 2–3 times/week.

The *in vitro* tests regarding the interaction of cells with the nanocarriers as well as the drug loaded nanocarriers cytotoxicity were carried out in a 2D monolayer culture system. In contrast, the drug loaded nanocarriers penetration in 3D micro tumors was evaluated using scaffold free 3D culture systems (spheroids). To obtain the conventional 2D culture systems, HT-29 colon cancer cells were seeded at an initial cell density of 2.5 × 10^4^ cell/cm^2^ on cell culture plastic surfaces. These cultures were allowed to adhere for 24 h before treatment. The spheroids were obtained as previously described (Gǎlǎṭeanu et al., 2016) in 4 days post-seeding of 5 × 10^3^ cells/drop and treatments were applied in the fifth day of culture.

#### Interaction of cells with PHBHV nanocarriers

SEM and AFM microscopic analyses were employed to investigate the interaction between HT-29 colon cancer cells and the PHBHV empty and drug loaded nanocarriers. In this view, HT-29 colon cancer cells were seeded in monolayers on cover glasses and allowed to attach for 24 h. Subsequently, the samples were treated for 24 h with the PHBHV nanoparticles and then fixed with a 2.5% glutaraldehyde solution. Upon fixation, the samples were washed and imaged with AFM. For the SEM analysis, the samples were air dried and sputtered with a thin layer of gold (5 nm) before inspection. A sample of untreated cells served as control in both cases.

#### Cell viability

The MTT assay was employed to investigate the HT-29 colon cancer cells viability (Mosmann, 1983). Briefly, the HT-29 colon cancer cells monolayers were treated for 2, 6, and 24 h with Silymarin (100 μg/ml), empty and Silymarin loaded PHBHV nanocarriers. At each time point the culture medium was discarded and the monolayers were washed with PBS. The samples were next incubated for 4 h at 37°C in a 1 mg/ml MTT solution prepared in DMEM. The formazan crystals were consequently dissolved in DMSO and the absorbance of the resulting solutions was measured at 550 nm using an Appliskan Thermo Scientific spectrophotometer. An untreated control was prepared under identical conditions and used as reference.

#### Silymarin loaded PHBHV nanocarriers cytotoxic potential on HT-29 cancer cells

The cytotoxic potential of the Silymarin loaded PHBHV nanoparticles on HT-29 colon cancer cells was investigated by the spectrophotometric evaluation of the Lactate Dehydrogenase (LDH) activity in the culture media. Briefly, after 2, 6, and 24 h of HT-29 monolayers exposure to Silymarin (100 μg/ml), unloaded and Silymarin loaded nanocarriers, the culture medium was harvested and mixed with the components of the TOX-7 kit (LDH based *in vitro* toxicology assay kit, Sigma Aldrich Co., Germany) according to the manufacturers' instructions. After a short incubation of 20 min at room temperature in the dark, the absorbance of the samples was determined at 490 nm using an Appliskan Thermo Scientific spectrophotometer. The same protocol was performed using culture media harvested from untreated monolayers.

#### *In vitro* PHBHV nanoparticle penetration potential into 3D micro tumors

*The Live/Dead fluorescence assay* was employed to image both the living and the dead cells under treatment conditions in the 3D culture systems. The Live/Dead fluorescence-based kit used (Invitrogen, Life Technologies, Foster City, CA, USA) contains two color dyes: calcein AM (green) and ethidium bromide (red) in order to discriminate at the same time the population of live cells from that of the dead cells. Briefly, 3D HT-29 spheroids exposed for 24 h to treatment with Silymarin (100 μg/ml), empty and Silymarin loaded PHBHV nanocarriers were incubated at room temperature in the dark for 20 min with the staining solution prepared according to the manufacturers' instructions. An untreated sample was prepared identically and used as a reference. The spheroids were then washed with PBS and imaged using an Olympus IX73 inverted fluorescence microscope. Images were captured using the CellSense Imaging Software (Olympus, Germany).

### Statistical analysis

All the spectrophotometric data were statistically analyzed using the GraphPad Prism 3.03 Software, one-way ANOVA, Bonferroni test. Data are presented as the average of three replicates (mean ± standard deviation).

## Results

### PHBHV nanocarriers synthesis

In this paper, Silymarin loaded PHBHV nanoparticles were prepared via the nanoprecipitation method using a PVA aqueous solution as stabilizer for the polyester solution (organic phase) (Galindo-Rodriguez et al., 2004). The particle size was controlled through a comparative study of the effect of critical factors such as polymer concentration (organic phase), stabilizer concentration (aqueous phase), and ratio between the two phases during nanoparticle preparation. In order to obtain smaller nanoparticle size which was crucial for our studies, the optimal initial polymer and stabilizer concentration was determined to be 2% w/w. The ratio between the organic and the aqueous phase was set at 1:10 (organic: aqueous phase volume).

### SEM and AFM analysis

**Figure 2:**
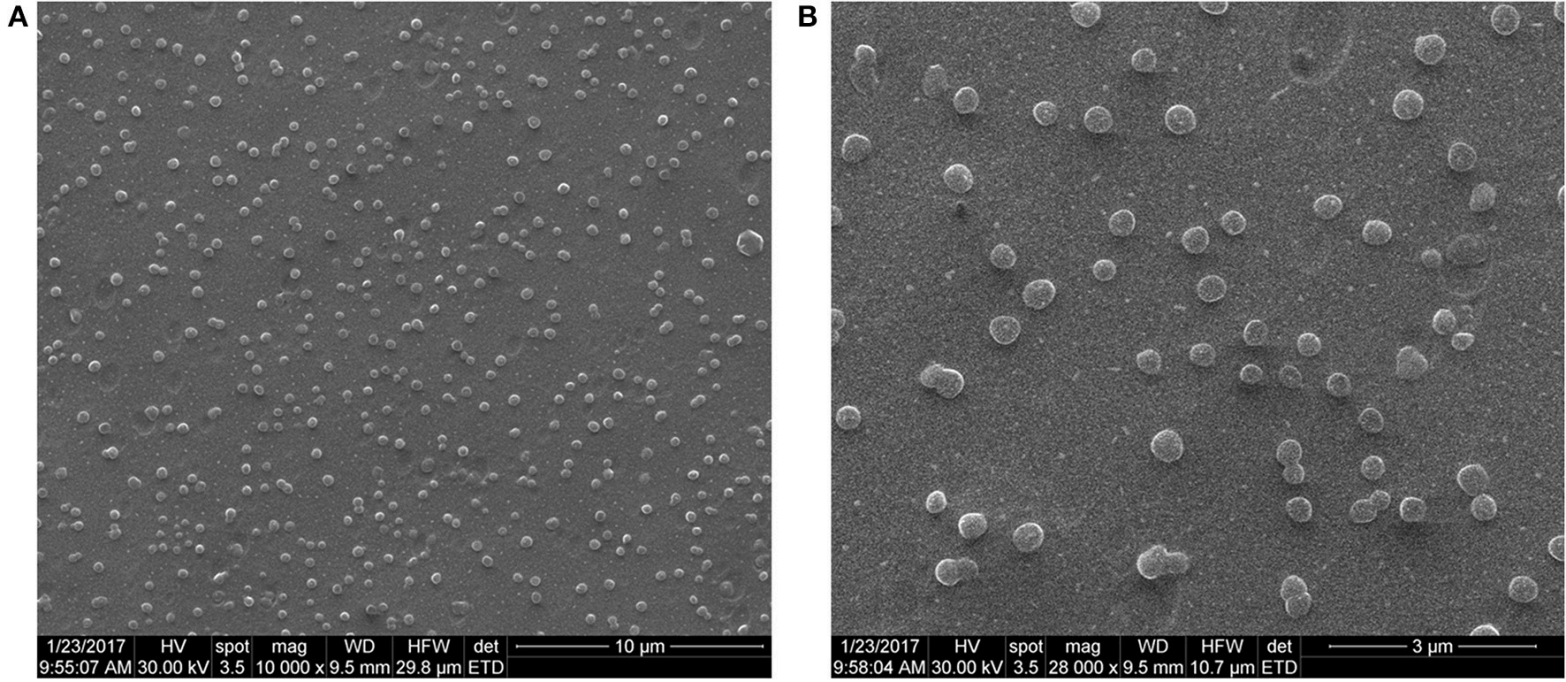
SEM microphotographs of the PHBHV nanoparticles (**A**—10,000X, **B**—28,000X).

**Figure 3:**
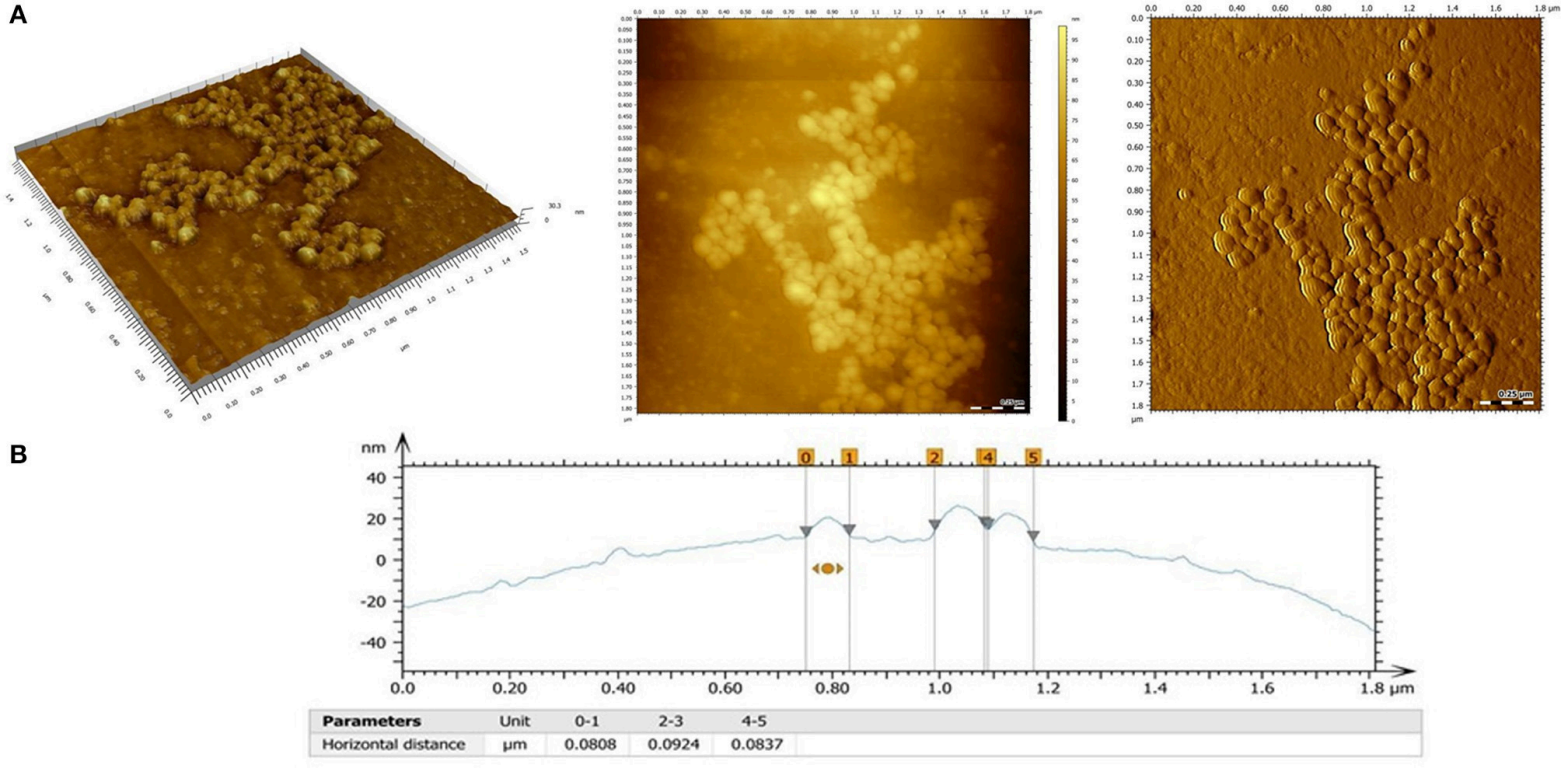
**(A)** AFM images of the PHBHV nanoparticles: 3D topography image (left), 2 D topography (center), deflection image (right); **(B)** Surface profile and size profile of the PHBHV nanoparticles as measured by AFM.

Both SEM (Figures 2A,B) and AFM (Figures 3A,B) investigations proved that by using the nanoprecipitation method at the aforementioned conditions, we were able to obtain well individualized PHBHV nanoparticles and control their size and size distribution (Galindo-Rodriguez et al., 2004). The low and high magnification SEM images (Figures 2A,B) revealed high number of nanoparticles, which displayed narrow size distribution of around 100–150 nm. Furthermore, the micrographs revealed that the nanoparticles were well-individualized and possessed a round shape morphology characterized by clean surface indicating the absence of trapped stabilizer mass.

2D and 3D AFM measurements (Figure 3A) revealed a spherical morphology for the PHBHV nanoparticles, characterized by individualized nanoparticles with a narrow size distribution and absence of aggregation even at high concentrations.

Additionally, by evaluating the obtained AFM surface profile (Figure 3B), a diameter of about 80–100 nm was determined for the PHBHV nanoparticles.

### Silymarin drug release from PHBHV nanocarriers

**Figure 4:**
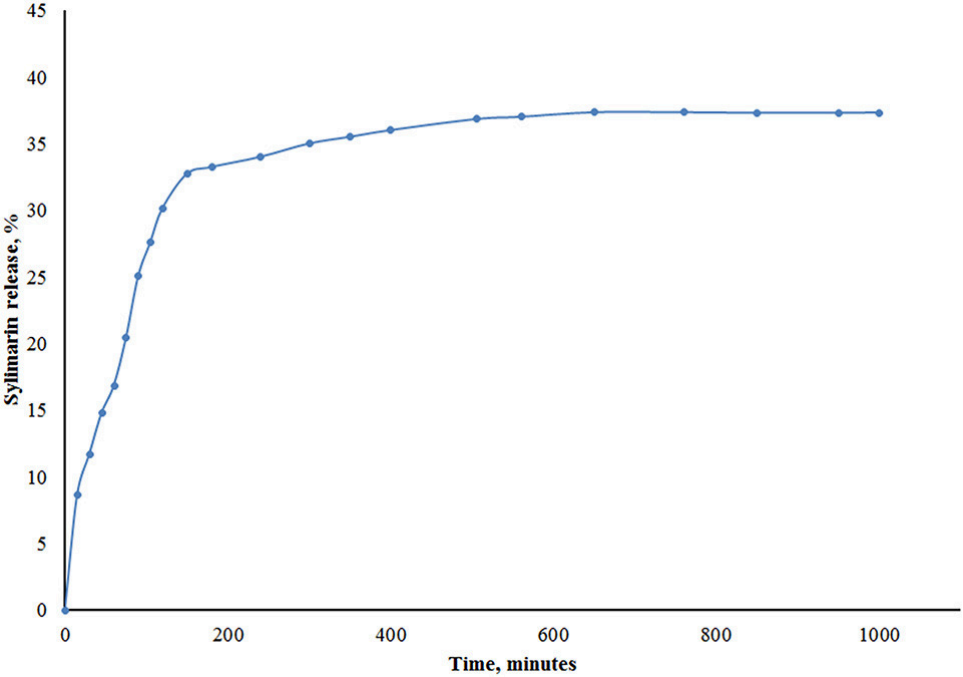
*In vitro* release profile of Silymarin from loaded-PHBHV nanoparticles.

The drug release profile of the Silymarin loaded nanoparticles in PBS (Figure 4) showed that 32% of the encapsulated Silymarin was released within the first 2.5 h reaching a maximum of approximately 38% after 20 h. The EE and DC were determined as previously described and were found to be 61.1 and 0.75%, respectively.

### PHBHV nanocarriers *in vitro* interaction with colon cancer cells

**Figure 5:**
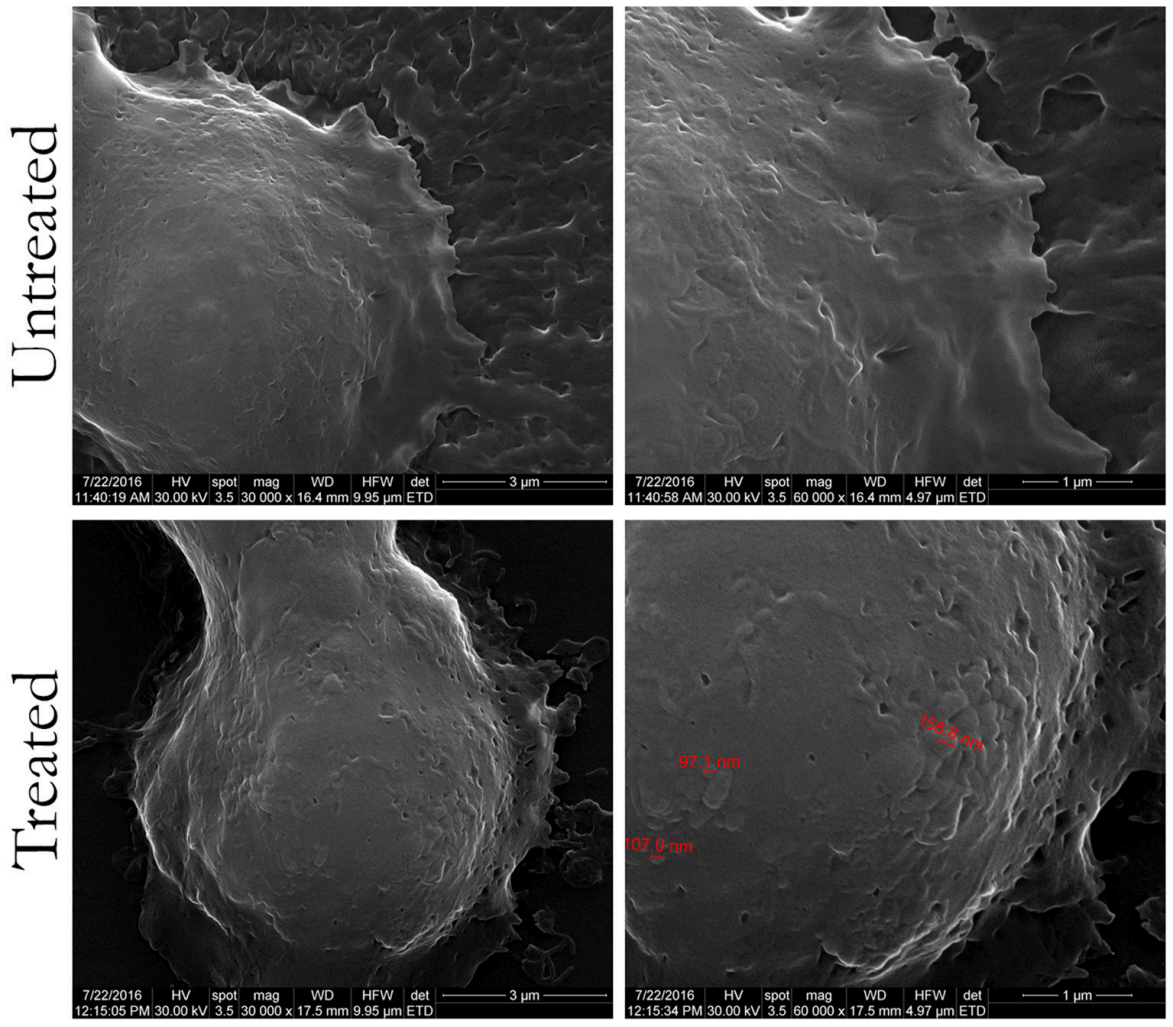
Scanning electron microscopy images of untreated HT-29 colon cancer cells and treated with PHBHV nanoparticles (magnification of: 16,000X/left, 30,000X/center, and 60,000X/right).

SEM and AFM were also employed to investigate the interaction between HT-29 colon cancer cells and the PHBHV nanocarriers. As shown in Figure 5, SEM revealed the presence of PHBHV nanoparticles of a diameter of about 100 nm on the surface of the HT-29 colon cancer cells.

**Figure 6:**
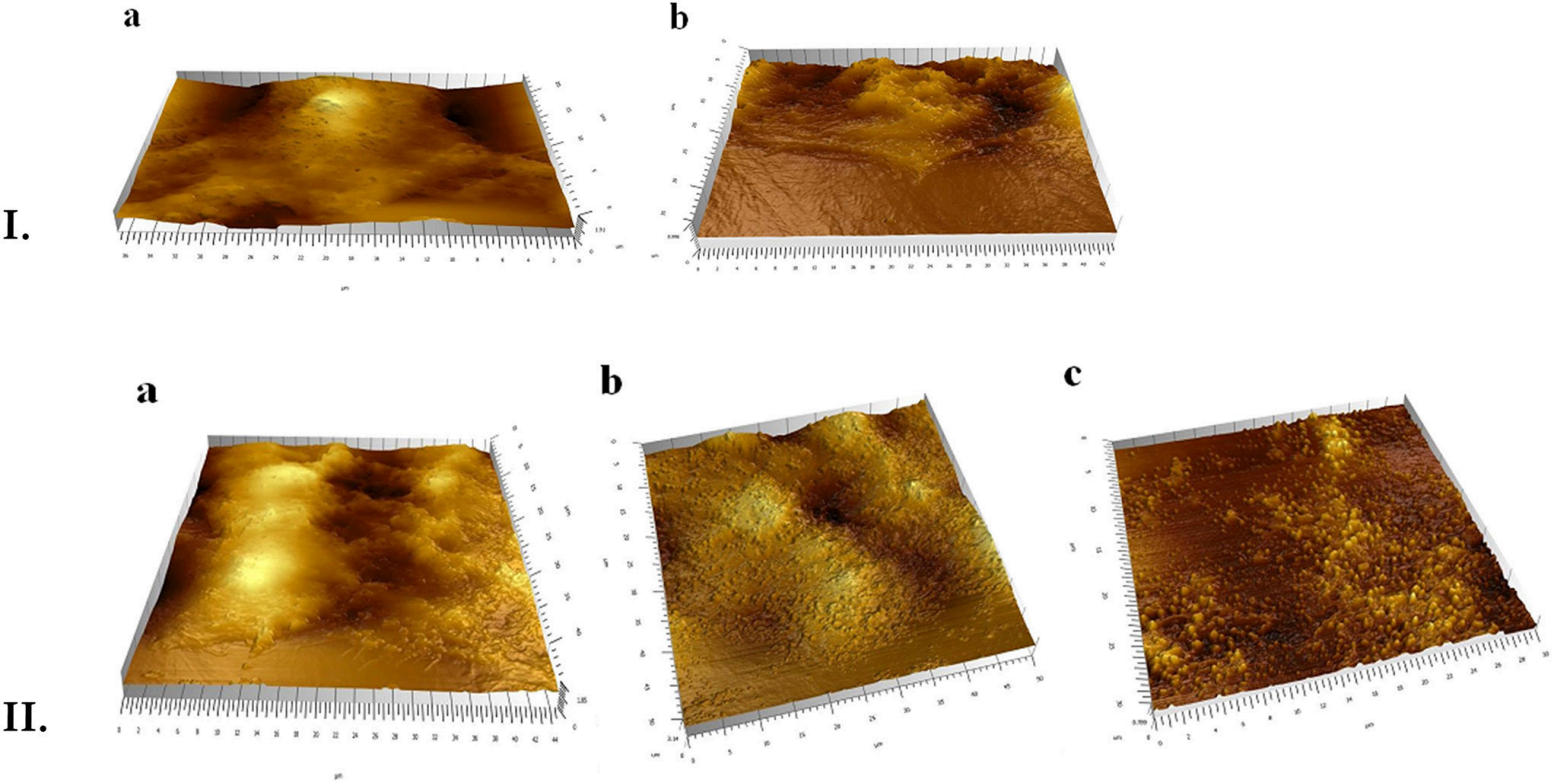
**(I)** AFM evaluation of the HT-29 monolayers: a, untreated; b, treated with PHBHV nanoparticles; **(II)** AFM 3D images: a, untreated HT-29 cells after mechanical breaking of their membrane; b, cells treated with nanoparticles prior to membrane breaking: c, post membrane breaking release of the nanoparticles from the cells.

To observe specific behavior and biological processes of the HT-29 cells interacting with PHBHV nanoparticles, fast and accurate morphological techniques, such as AFM are required. A soft cantilever with a spring constant of about 0.06 N/m was used during these experiments, as its flexibility is vital for cell imaging in order to allow high bending when in contact with surface features. At the same time, the tip should not submit a high resistance or deform these surface features. As revealed in Figure 6I, the presence of PHBHV nanoparticles on the surface of HT-29 colon cancer cells induced morphological modification of the surface topography, visualized by the presence brighter areas. Consequently, these surface features could be attributed to nanoparticle accumulation, which tend to get relief through the cell membrane, deforming thus the cell structure.

In order to evaluate the uptake of the PHBHV nanoparticles by the HT-29 colon cancer cells, a mechanical test was performed. More specifically, upon bringing the tip of the instrument into contact with the cell surface, the pressing force was increased until the cell membrane was pierced. This technique enables the study of individual cells and allows imaging of the surface of the sample as well as of the interaction of the sample under study with the tip. As shown in Figure 6II, nanoparticles and nanoparticle aggregates were released through this mechanical breaking of the cell membrane. A similar experiment performed on untreated cells did not afford any nanoparticles upon membrane breaking. Furthermore, AFM images indicated that most of the nanoparticles were entrapped within the cells, otherwise they would have been drawn away by the tip force (Figure 6I).

### HT-29 cells viability after treatment with unloaded and Silymarin loaded PHBHV nanocarriers

**Figure 7:**
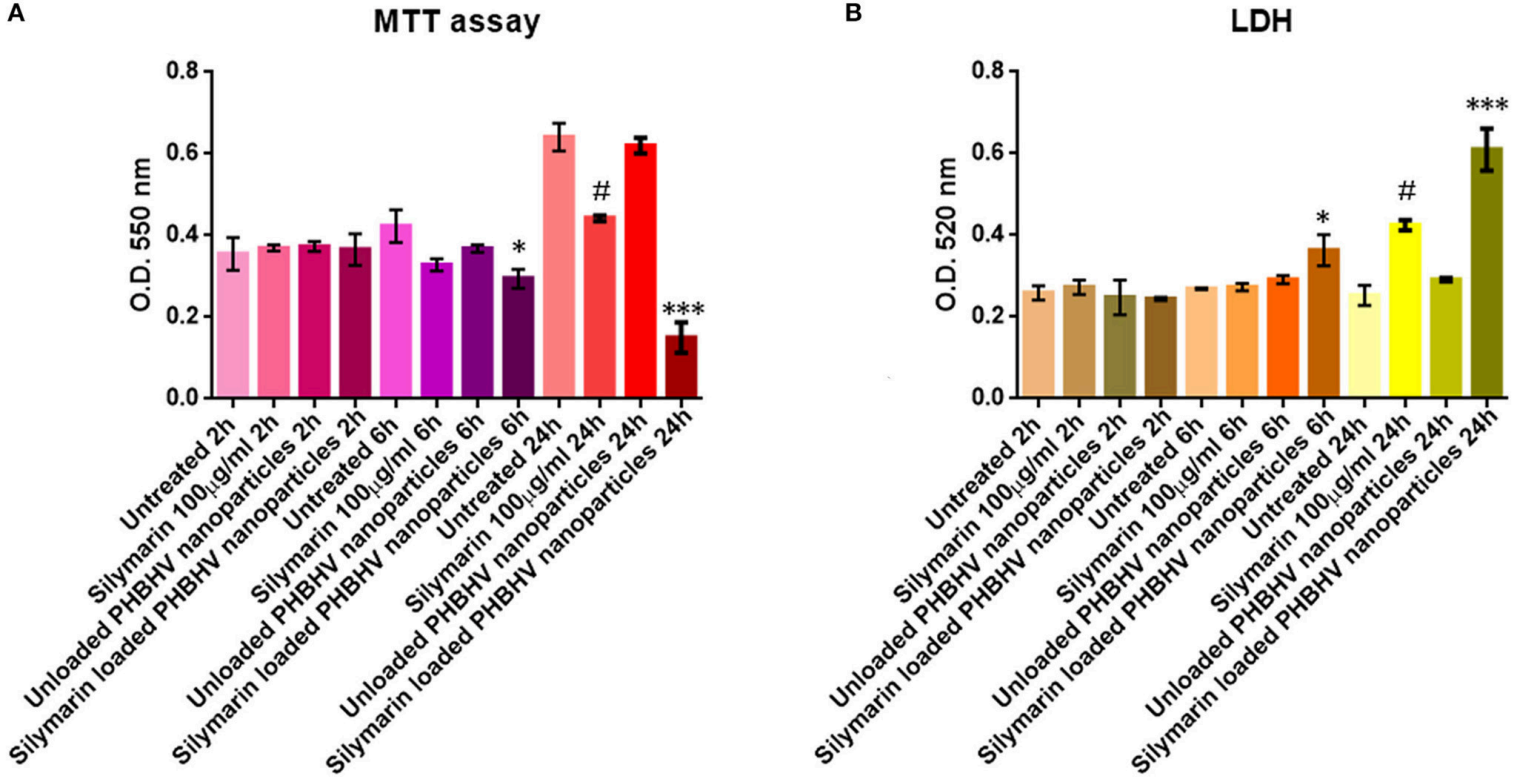
Graphic representation of spectrophotometric data obtained by the MTT **(A)** and LDH **(B)** assay. ^*^p < 0,05 Silymarin loaded PHBHV nanocarriers vs. untreated sample after 6 h. ^***^p < 0.001 Silymarin loaded PHBHV nanocarriers vs. untreated sample after 24 h. #*p* < 0.05 Silymarin 100 μg/ml vs. untreated sample at 24 h.

The viability of HT-29 colon cancer cells was investigated after 2, 6, and 24 h of treatment with Silymarin (100 μg/ml) and the PHBHV nanoparticles using the MTT quantitative assay (Mosmann, 1983). The obtained data were statistically analyzed using the GraphPadPrism Software and are graphically represented in Figure 7A. No significant differences were observed between the Silymarin, PHBHV treated samples and the untreated control after 2 h of treatment. However, after 6 h of treatment with the Silymarin loaded PHBHV nanocarriers, the viability of the HT-29 colon cancer cells decreased significantly as compared to that of the untreated sample (*p* < 0.05). Moreover, after 24 h of exposure, there were no significant viability differences registered between the untreated cells and the cells exposed to unloaded PHBHV nanoparticles. In contrast, cellular viability decreased dramatically after 24 h of treatment with the Silymarin loaded PHBHV nanocarriers as compared with both the untreated reference sample and with the sample treated with the unloaded PHBHV nanoparticles (*p* < 0.001). Silymarin treatment induced a moderate cell viability decrease at 24 h of treatment (*p* < 0.05).

### Evaluation of the nanocarriers cytotoxic potential

The results obtained from the investigation of LDH activity in the culture media of HT-29 monolayers treated for 2, 6, and 24 h with Silymarin, unloaded and Silymarin loaded nanocarriers and the reference cells, were in full accordance with the MTT cell viability test. As shown in Figure 7B, no significant differences were found between treated and untreated samples after 2 h. Furthermore, it is clearly demonstrated that the unloaded PHBHV nanoparticles did not exert any cytotoxic effect on HT-29 colon cancer cells, while Silymarin loaded PHBHV nanocarriers treatment significantly increased LDH activity in the culture media after 6 (*p* < 0.05) and 24 h (*p* < 0.001). Silymarin alone moderately increased LDH activity in the culture media at 24 h of treatment (*p* < 0.05).

### PHBHV nanocarriers penetration potential into 3D micro tumors

The nanoparticle penetration potential into 3D micro tumors was investigated after a 24 h treatment of HT-29 spheroids with Silymarin (100 μg/ml), unloaded PHBHV nanoparticles and Silymarin loaded nanocarriers by double staining the live and the dead cells with the fluorescent dyes calcein and ethidium bromide respectively. An untreated sample was also studied as reference. For each sample 10 spheroids were analyzed in fluorescence microscopy using CellSense Imaging Software and an Inverted Olympus IX73 microscope with fluorescence modulus.

**Figure 8:**
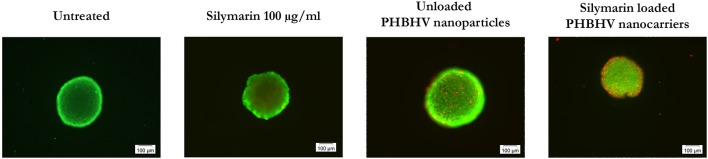
Fluorescence microscopy images of live (green fluorescence) and dead (red fluorescence) HT-29 colon cancer cells in scaffold free 3D micro tumors.

As shown in Figure 8, all samples displayed bright green living cells. Dead cells were observed in all the treated samples, with the amount of the dead cells in the sample treated with the Silymarin loaded PHBHV nanocarriers being highly increased, probably as a result of the treatment's cytotoxic action. Interestingly, the dead cells in the latter sample were observed both on the edge of the 3D micro tumor as well as inside its mass.

## Discussion

Colo-rectal cancer is an important cause of mortality and morbidity globally and one of the most prominent causes of neoplastic mortality (Shahidi and Cheung, 2016). Given its increasing incidence, Colo-rectal cancer management has gained much attention in research focusing both to diminish risks and to control progression of the disease. In this view, new research has been developed based on the observation that certain types of diets, particularly those based on abundant vegetable and fruit intake may present protective potential against risk factors for various cancer types. Consequently, studies on various herbs and plants with medicinal use have given rise to the hypothesis regarding their cancer chemo-preventive potential. Positive results in this direction have been added to the chemo-preventive pharmacological action specific to phyto-chemicals in certain concentrations of the invaluable advantage of lack of toxicity (Scarpa and Ninfali, 2015).

The main drawback in the study and use of these active compounds is their limited bioavailability in the human body once administered caused by their poor water solubility. In fact, more than 40% of such drugs show low water solubility and consequently, their administration is limited to oral delivery, local injection or surface retention (Cheng et al., 2007). In this context, loading such drugs on nanosized polymeric shuttles is widely investigated since it could increase drug bioavailability and offer alternative administration routes such as intravenous injection. Moreover, current studies in oncology have approached new strategies to increase treatment outcomes and lower the overall toxic effect of chemo-therapeutic agents by developing nanosized targeting and drug delivery systems. Additionally, many relevant studies show that nanoparticles can accumulate in tumors after intravenous administration while their biodistribution is largely determined by their physical and biochemical properties which can be highly tailored (Chenga et al., 2007; Baran et al., 2013; Guo et al., 2013; Mua and Fenga, 2013).

In this study, we present the development of new biodegradable and biocompatible nanosized shuttles for Silymarin targeted delivery in cancer cells. The design of these novel carrier nanoparticles was based on natural PHBHV polymers and tested in terms of biological properties such as cellular uptake potential, cytotoxicity, and 3D penetrability against a colon cancer cell line (HT-29) as the *in vitro* culture model. The PHBHV nanoparticles were easily obtained via the nanoprecipitation method. The controlled and slow addition of the polymer solution to the PVA/drug aqueous solution played a key role for the formation of small nanoparticles, a fact attributed to the controlled diffusion of the polymer solution through the aqueous phase. Our results clearly demonstrate that controlled nanoprecipitation is the appropriate method for obtaining nanoparticles with diameters smaller than those obtained through other methods of nanoparticle preparation (Galindo-Rodriguez et al., 2004).

The drug release profile and the EE results show that part of the Sylimarin remained entrapped within the PHBHV nanoparticles. The newly developed nanoparticles exhibit a core-shell structure with a hydrophobic core formed by the PHBHV and a hydrophilic shell formed by the PVA macromolecules. The nanoprecipitation method used to prepare these nanocarriers involves direct solubilization of the drug in the polyester solution leading thus to nanoparticles with the drug molecules distributed both in the core and the shell of the nanoparticle. The drug release curve (Figure 3) shows a fast release about 32% in the first 2.5 h which may be attributed to the release of the drug molecules adsorbed on the nanoparticle surface while the remaining drug, released up to the 38% plateau, may stem from the drug molecules physically entrapped within the hydrophilic outer shell. At the same time, the hydrophilic PVA macromolecules that form the shell could also solubilize within the aqueous medium and release part of the drug. The remaining drug is most probably entrapped within the core of the nanoparticles and can be released upon biodegradation. Since PBS is not a sustainable medium and cannot imitate biological conditions or highlight the entire drug release profile of the PHBHV loaded nanoparticles an *in vitro* release study would provide no further data (Chaput et al., 1995; Cheng et al., 2003). The potential participation of enzymes found in the human body in the hydrolysis of P3HB discussed in previous studies in the literature suggests that in the presence of lysozyme, full *in vitro* biodegradation occurs in about 40 days (Cheng et al., 2003). However, *in vivo* experiments are still needed for further clarification of P3HB and PHBHV biodegradation mechanisms and kinetics. Interestingly, while interacting with HT-29 cells the drug loaded PHBHV nanoparticles caused a pronounced increase in the amount of the dead cells, which could be attributed to a possible PHBHV biodegradation accompanied by drug release from the core.

The morphology, size, size distribution, and interaction of the nanoparticles with the cells were investigated using SEM and AFM. Additionally, SEM allowed studying the interaction between cells and nanoparticles and imaging PHBHV nanoparticles of about 100 nm diameter in contact with HT-29 cells. Furthermore, AFM enabled us to determine that the PHBHV nanoparticles reached the intracellular compartment. Considering these results, AFM played an indispensable role in the imaging and manipulation of HT-29 colon cancer cell samples. However, the cellular uptake mechanisms should be further studied in order to be fully elucidated.

The study using the MTT assay revealed no significant differences in HT-29 cell viability when comparing an untreated monolayer and a sample exposed to unloaded PHBHV nanoparticles for 2, 6, and 24 h, indicating a low cytotoxicity for the PHBHV nanocarriers on HT-29 malignant cells. However, the same test showed that HT-29 cell viability decreased significantly after 6 and 24 h of treatment with Silymarin loaded nanocarriers, a fact that should be attributed to Silymarins' cytotoxic effect on colon cancer cells. These findings were strongly supported by the results obtained after investigating the potential cytotoxic effects on cells via the LDH assay. Furthermore, the time lag in cell viability decrease might be correlated both with the cellular nanocarrier uptake process and with Silymarin release from the carriers. Our results are in accordance with recent studies which revealed that Silibinin/Silymarin induces apoptosis in HT-29 colon cancer cells through up regulation of non-steroidal anti-inflammatory drug activated gene-1 (Woo et al., 2014). Additional investigations of the mechanisms responsible for anti-cancer and pro-apoptotic capacity of plant polyphenols have produced evidence of their ability to stop the cell-cycle by inactivation of the CDC2 cell cycle regulator as well as induction of cyclins A and E (Larrosa et al., 2003).

In previous work, we demonstrated the crucial importance of using 3D culture models for *in vitro* studies in oncology (Barzaghi et al., 1990). Regardless of the 3D system design (scaffold free or cells/scaffold bioconstructs), these multicellular micro tumors were developed to better mimic *in vitro* the *in vivo* environment and conditions. For example, the third dimension is crucial for the investigation of the delivery systems penetrability potential. Taking these facts into account, in our current study we treated HT-29 3D multicellular tumor spheroids for 24 h with unloaded PHBHV nanoparticles and with Silymarin loaded nanocarriers. Upon fluorescent staining of both living and dead cells, we observed an increased number of dead cells present in the sample exposed to Silymarin carriers, clearly demonstrating the shuttle's penetration into the spheroid followed by cellular uptake and drug release.

## Conclusions

In conclusion, we have designed and constructed biocompatible and biodegradable PHBHV nanoparticles of spherical shape and about 100 nm diameter by the nanoprecipitation method. These nanoparticles were tested for their capacity to deliver Silymarin into HT-29 colon cancer cells. The PHBHV nanoparticles did not influence HT-29 cells viability or exert any cytotoxic effects on the cells. More importantly, the Silymarin loaded PHBHV nanocarriers significantly decreased HT-29 cell viability after 6 and 24 h of treatment. Moreover, by using a HT-29 multicellular spheroids culture model we were able to confirm the ability of the PHBHV nanocarriers to penetrate 3D structures and to deliver the drug. Current studies are focused on exploring the mechanisms of Silymarin loaded nanoparticles action against cancer cells.

## Author contributions

CZ, PS, HI, OG, PL, and CN designed the PHBHV nanoscaled drug delivery system. IR and CZ synthetized the nanoparticles and performed all the studies regarding the drug uptake and release as well as the AFM analysis. EV performed the SEM analysis. AH, BG, and MC designed and performed the *in vitro* experimental evaluation of the systems cytotoxicity and cellular uptake. BG, CZ, MS, and KV drafted the manuscript. AT was responsible for the coordination of the study and assured a good collaboration between the authors.

### Conflict of interest statement

The authors declare that the research was conducted in the absence of any commercial or financial relationships that could be construed as a potential conflict of interest.

## Abbreviations

AFM: atomic force microscopy
DC: drug content
DMEM: Dulbecco's modified Eagle's medium
EDS: energy dispersive spectrometer
EE: encapsulation efficiency
FBS: fetal bovine serum
FEG: field emission gun
LDH: lactate dehydrogenase
MTT: 3-(4,5-dimethylthiazol-2-yl)-2,5-diphenyltetrazolium bromide
PBS: phosphate buffered saline
PHBHV: Poly(3-HydroxyButyrate-co-3-HydroxyValerate)
PVA: polyvinyl alcohol
SEM: scanning electron microscopy.
